# Aerobic and facultative anaerobic *Klebsiella pneumoniae* strains establish mutual competition and jointly promote *Musca domestica* development

**DOI:** 10.3389/fimmu.2023.1102065

**Published:** 2023-02-17

**Authors:** Kexin Zhang, Shumin Wang, Dawei Yao, Xinyu Zhang, Qian Zhang, Wenjuan Liu, Ying Li, Yansong Yin, Sha An, Ruiling Zhang, Zhong Zhang

**Affiliations:** ^1^ School of Basic Medical Science, Shandong First Medical University, Shandong Academy of Medical Sciences, Taian, Shandong, China; ^2^ Collaborative Innovation Center for the Origin and Control of Emerging Infectious Diseases, Shandong First Medical University, Shandong Academy of Medical Sciences, Taian, Shandong, China; ^3^ School of Life Science, Shandong First Medical University, Shandong Academy of Medical Sciences, Taian, Shandong, China; ^4^ Shandong Institute of Endocrine and Metabolic Diseases, Shandong First Medical University, Jinan, Shandong, China; ^5^ School of life Science, Weifang Medical University, Weifang, Shandong, China; ^6^ Medical Science and Technology Innovation Center, The First Affiliated Hospital of Shandong First Medical University, Jinan, Shandong, China

**Keywords:** intestinal flora, housefly, aerobic bacteria, facultative anaerobic bacteria, *K. pneumoniae*, phage

## Abstract

**Introduction:**

The gut microenvironment in housefly harbors a rich and diverse microbial community which plays a crucial role in larval development. However, little is known about the impact of specific symbiotic bacteria on larval development as well as the composition of the indigenous gut microbiota of housefly.

**Methods:**

In the present study, two novel strains were isolated from housefly larval gut, i.e., Klebsiella pneumoniae KX (aerobe) and K. pneumoniae KY (facultative anaerobe). Moreover, the bacteriophages KXP/KYP specific for strains KX and KY were used to analyse the effects of K. pneumoniae on larval development.

**Results:**

Our results showed that dietary supplementation with K. pneumoniae KX and KY individually promoted housefly larval growth. However, no significant synergistic effect was observed when the two bacterial strains were administered in combination. In addition, using high-throughput sequencing, it was demonstrated that the abundance of Klebsiella increased whereas that of Provincia, Serratia and Morganella decreased when housefly larvae received supplementation with K. pneumoniae KX, KY or the KX-KY mixture. Moreover, when used combined, K. pneumoniae KX/KY inhibited the growth of Pseudomonas and Providencia. When the abundance of both bacterial strains simultaneously increased, a balance in total bacterial abundance was reached.

**Discussion:**

Thus, it can be assumed that strains K. pneumoniae KX and KY maintain an equilibrium to facilitate their development in housefly gut, by establishing competition but also cooperation with each other to maintain the constant composition of gut bacteria in housefly larvae. Thus, our findings highlight the essential role of K. pneumoniae in regulating the composition of the gut microbiota in insects.

## Introduction

The microorganisms found in the gut of insects form a stable community and affect the physiological functions of the host (1–3). Moreover, it is widely known that bacteria in the gut of insects play a dominant role during insect development and reproduction, but also in the digestion, since they provide important nutrients to the host (4, 5). In addition, certain microorganisms shape the competence of mosquito female vectors by modulating their immune response (6).

However, an antagonistic relationship between bacteria in the gut of housefly larvae has been suggested, which likely affects insect growth and development. For instance, *Enterobacter hormaechei* can inhibit the growth of harmful bacteria in the gut of the housefly larvae, such as *Pseudomonas aeruginosa*, *Providencia stuartii* and *Providencia vermicola*, and promote the reproduction of beneficial bacteria (7). In *Anopheles* mosquitoes, *Enterobacteriaceae* could interfere with parasite development prior to invasion in the host midgut epithelium, thus protecting the host from *Plasmodium* infection (8, 9). Moreover, it has been shown that substrate-associated microorganisms affect black soldier fly larval performance by altering larval microbiota (10). In addition, certain intestinal bacteria can affect the transmission of dengue virus, increase the infection of vector pathogens (11) and interact with pathogenic fungi to promote mosquito infection (12). It has been demonstrated that viral infection in *Aedes* (13) and *Anopheles* mosquitoes (14) is increased by the presence of *Serratia odorifera* in the midgut. For instance, *S. odorifera* in the midgut of the mosquito *Aedes aegypti* increases its susceptibility to dengue type-2 virus by blocking the prohibitin molecule found on the surface of host midgut (13).

Houseflies (*Musca domestica*) belong to the family Muscidae, which harbors a great number of species. Houseflies have an expected life span of approximately 30 days, during which time four distinct developmental stages unfold: egg, larvae, (pre) pupa, and adult (15). The gut of housefly larvae provides a suitable environment for microbial colonization, and it has been shown that gut bacteria play an important role in the regulation of both physiological and pathological processes of housefly (15). However, the regulatory mechanism by which specific bacteria influence the development of housefly larvae is poorly understood. Most studies on insect gut bacteria are based on the “total removal and replenishment” model, which interferes with untargeted bacteria in the intestine (16–18). Therefore, it is necessary to design precise and predictable approaches to target and control susceptible bacterial species in the gut microbiota in order to remodel it and study its functions.

Bacteriophages are viruses that specifically infect and parasitise bacteria. Phages can be used as efficient antibacterial agents that regulate indigenous bacterial populations (19). Previous studies have discussed the essential role of phages in the formation of bacterial communities and have highlighted their potential therapeutical value to animals (20–22) and plants (23–25). In contrast to antibiotics and bacterial inoculants (26), phages can invade bacterial cells with high specificity *via* specific cell surface receptors. Therefore, phages with the appropriate properties could be leveraged as powerful tools for targeting specific species.

Previous studies have revealed that phages can regulate insect gut bacterial community and re-establish health in insect models previously infected by bacteria (27, 28). Moreover, studies in animal models have yielded promising results. In addition, the bacteriophage BUCT610 could significantly improve the survival rate of *Galleria mellonella* larvae challenged with *Klebsiella pneumoniae* infection (29). Other studies have emphasized the therapeutic potential of phages in insects (30, 31). For instance, two phages showed activity against the strain *Klebsiella pneumoniae* ST13 and could replicate the decrease in bacterial titers in *G. mellonella* larvae, thus improving the survival of infected larvae (31). In another study, the *Bordetella bronchiseptica* phage vB_BbrP_BB8 could efficiently eliminate biofilm and prevent lethality of *B. bronchiseptica* in *G. mellonella* honeycomb moth larvae (30). Moreover, the interplay between phages and the gut microbiota is complex and may be affected by the host immune system (32–35), spatial structure within the tissue, nutrient availability (35) and local microbiota. However, experimental models established in previous studies are usually based on single bacteria-phage pairs in the natural environment. The interaction between phages and their targeted bacteria as well as its effect on housefly larval gut bacterial community are still poorly understood. Therefore, it is necessary to establish an insect model to better study the interaction between phages, targeted bacteria and gut microbiota.

In our previous research, a phage-targeted method was established to reduce the abundance of the strain *Pseudomonas aeruginosa* Y12 in housefly larval gut. Using *P. aeruginosa-*specific bacteriophage as a targeting tool, the effects of *P. aeruginosa* Y12 on housefly larvae growth, development, and gut microbiota composition were explored (36). Then our following research further found that bacteriophages can result in alteration of housefly gut community structure which further influenced housefly larval growth and development (37). Previous research showed that single bacteriophage could be used for function analysis of housefly gut microbes. However, the influence of combined application of two phages on housefly gut community, and function analysis of aerobic bacteria and facultative anaerobic bacteria in housefly intestine has been little reported till now. It is necessary to study multivalent phage cocktails on bacteria–phage interactions in insect guts in the future. In our experiment, function of bacteria was studied from aerobic and anaerobic states and using a single phage to reduce the host bacteria and using two-phage combinations to feed housefly larvae at the same time to study whether the combined application of phages has a synergistic effect. Thus, in order to expand the current understanding of the interplay between beneficial aerobic/acultative anaerobic gut bacteria and insect health, an insect gut model was proposed. The aerobic strain *Klebsiella pneumoniae* KX and the facultative anaerobic strain *K. pneumoniae* KY were isolated from housefly larval gut; simultaneously, the bacteriophage KXP of *K. pneumoniae* KX and the bacteriophage KYP of *K. pneumoniae* KY were isolated and used as specific antibacterial agents to regulate the abundance of *K. pneumoniae* KX/KY in housefly larval gut. Furthermore, the gut microbial community structure of larvae fed with *K. pneumoniae* KX/KY or bacteriophages KXP/KYP was analysed to elucidate the functional mechanism by which *K. pneumoniae* KX/KY impact housefly larval development. Our research revealed the important role of aerobic and facultative anaerobic *K. pneumoniae* played in the housefly aerobic midgut and the anaerobic hindgut. The results discussed herein highlight the potential of combined application of bacteriophages in future studies on insect gut microbiota.

## Results

### Characteristics of *Klebsiella pneumoniae*

**Figure 1:**
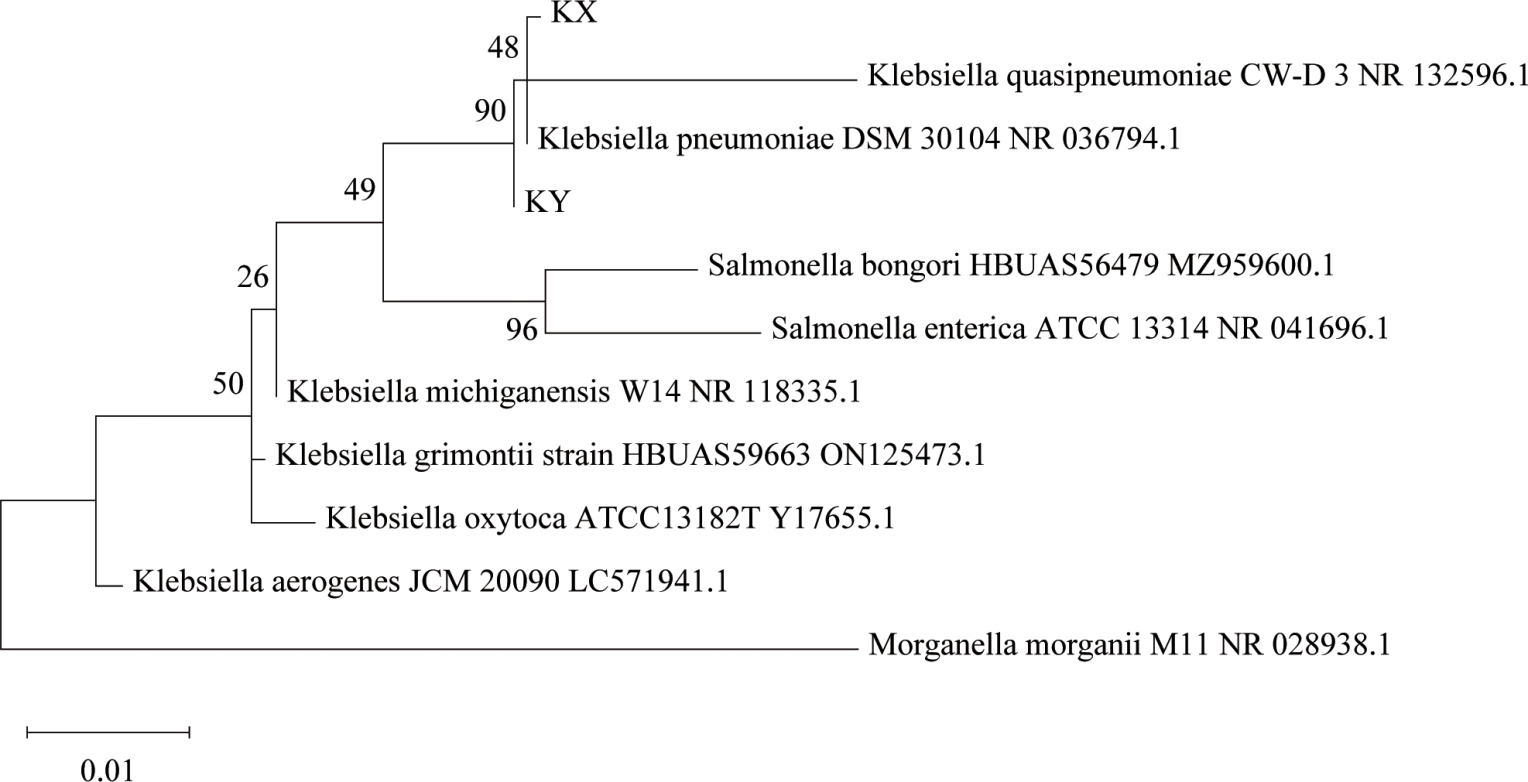
Phylogenetic analysis showing the evolutionary position of *Klebsiella pneumoniae* KX and KY. Phylogenetic position of KX and KY based on the 16S rRNA gene sequences. The phylogenetic analysis was performed using the Maximum Likelihood method in MEGA, and associated taxa clustered together in the bootstrap test of 2000 replicates.

Isolation and identification of *K. pneumoniae* from housefly larval intestines was achieved through the traditional isolation and culture method, the culturable aerobic *K. pneumoniae* KX was isolated from housefly larval intestines under an aerobic environment, and the culturable facultative anaerobic *K. pneumoniae* KY was isolated from housefly larval intestine under an anaerobic environment. The results showed that *K. pneumoniae* KY and *K. pneumoniae* KX clustered into different branches of the *K. pneumoniae* genus, indicating that they are two different strains of *K. pneumoniae* (**Figure 1**).

### Isolation of phages from sewage targeted against *K. pneumoniae*

**Figure 2:**
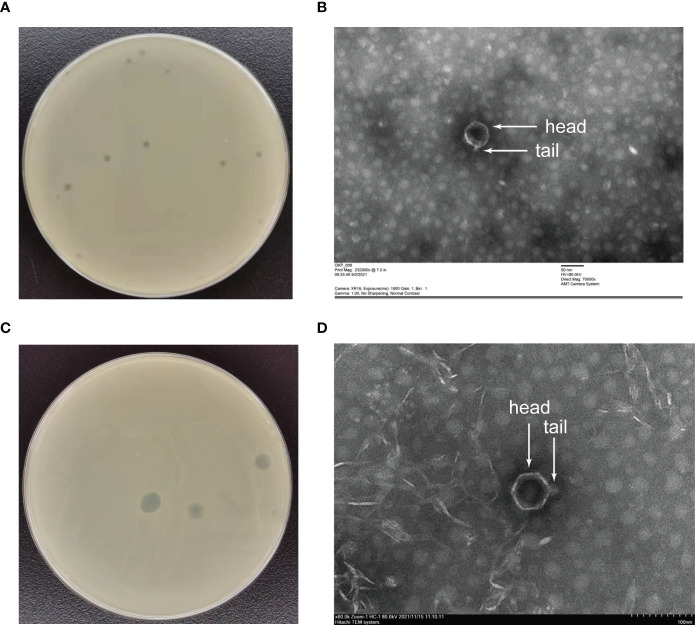
Morphology of phage KXP and phage KYP. **(A)** Morphology of phage KXP plaques in nutrient agar medium. The plaques of KXP are medium in size and transparent. **(B)** Electron micrograph of the negatively stained, purified phage KXP used in this study. **(C)** Morphology of phage KYP plaque in nutrient agar medium. The plaques of KYP are small and pinhole shaped, and there is a halo around the centre. **(D)** Electron micrograph of the negatively stained, purified phage KYP used in this study. KX, *Klebsiella pneumoniae* KX; KY, *Klebsiella pneumoniae* KY. Values are the means ± standard deviations from triplicates of each treatment.

**Figure 3:**
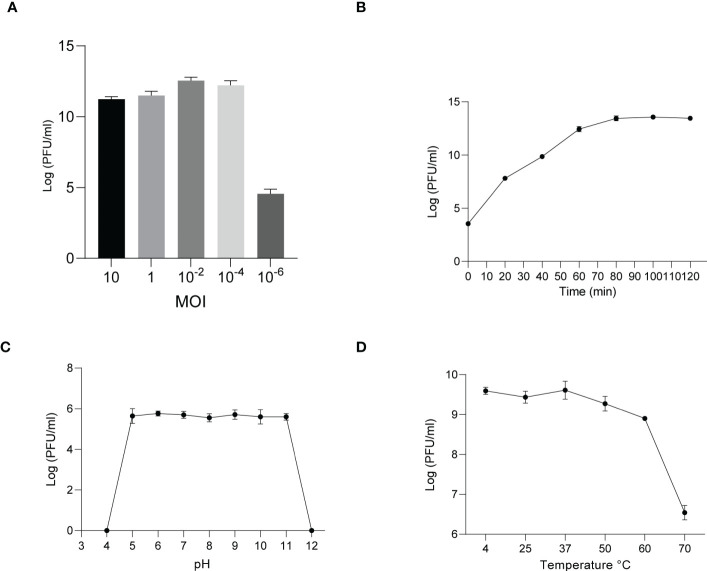
Analysis of the biological characteristics of phage KXP. **(A)** The MOI of phage KXP. The (*K*) *pneumoniae* KX strain was infected with phage KXP at various MOIs (10, 1, 10^-2^, 10^-4^, 10^-6^) and incubated at 37°C for 3.5 (h) When the MOI was 10^-2^, the titer reached the highest value (12.55 log PFU·mL-1). **(B)** One-step growth curve of phage KXP. Phage KXP has almost no latent period. The phage titer increased rapidly and stabilized at 80 minutes. The titer of the sample was measured at different time points. **(C)** The pH stability of phage KXP. Phage KXP is stable over a relatively wide range of pH values, and it (∼10^6^ PFU/mL) was incubated in PBS at different pH values ranging from 4 to 12 at 37°C for 1 (h) **(D)** The thermal stability of phage KXP determined at 4, 25, 37, 50, 60 and 70°C for 1 (h) Values are the means ± standard deviations from triplicates of each treatment.

The phages obtained from sewage were purified by a double-layer agar plate method and uniform plaque was produced on the double-layer plates (**Figures 2A–D**). Host specificity analysis revealed that phage KXP lysed only its susceptible bacteria *K. pneumoniae* KX and had no apparent impact on the other commensal bacteria isolated from the intestine of housefly larvae, including *K. pneumoniae* KY, *Providencia sneebia DSM* 19967, *Pseudocitrobacter faecalis*, *Morganella morganii*, *Enterobacter hormaechei*, *Enterococcus casseliflavus*, *Providencia. stuartii* and *Enterococcus faecalis* (**Table S1**). It was observed by transmission electron microscopy that the head of phage KXP has a typical icosahedral structure and short tail, which is consistent with the characteristics of *Podoviridae* (**Figure 2B**). Phage KXP had almost no latent period and tended to become stable after a sharp increase in titer, and the burst size was 3.73 × 10^13^/3.80 × 10^3^ = 9.82 × 10^9^ particles/infected cell (**Figure 3B**). When the multiplicity of infection (MOI, the ratio of the number of phages to the number of host bacteria) was 10^–2^, the phage KXP titer reached the highest level, approximately 12.55 log plaque forming unit (PFU) mL-1, indicating that the optimal multiplicity of infection (OMOI) (OMOI, the highest phage titration of the proportion of phage and its host) of phage KXP was 10^-2^ (**Figure 3A**). The phage could withstand a pH 5-11 environment for 1 h (**Figure 3C**). In addition, it withstood a temperature from 4°C to 60°C for 1 h (**Figure 3D**). In a certain range of temperature and pH, the phage KXP shows good stability. Genome sequencing analysis revealed that the genome of KXP had a total length of 42,946 bp, and the average GC content was 54.2%, the number of protein-coding genes is 45, the gene length/genome ratio is 0.885 (**Figure S1A**, **Table S2**).

**Figure 4:**
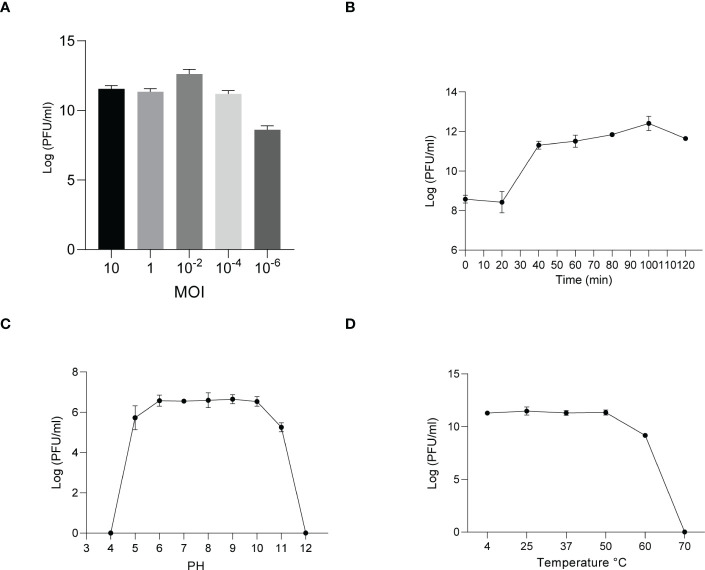
Analysis of the biological characteristics of phage KYP. **(A)** The MOI of phage KYP. The (*K*) *pneumoniae* KY strain was infected with phage KYP at various MOIs ((10, 1, 10^-2^, 10^-4^, 10^-6^) and incubated at 37°C for 3.5 **(**h) When the MOI was 10^-2^, the titer reached the highest value (12.61 log PFU mL-1). **(B)** One-step growth curve of phage KYP. Phage KYP has a latent period of 20 min. The phage titer increased rapidly and reached the highest value in 100 minutes. The titer of the sample was measured at different time points. **(C)** The pH stability of phage KYP. Phage KYP is stable over a relatively wide range of pH values, and it (∼10^6^ PFU/mL) was incubated in PBS at different pH values ranging from 4 to 12 at 37°C for 1 (h) **(D)** The thermal stability of phage KYP determined at 4, 25, 37, 50, 60 and 70°C for 1 (h) Values are the means ± standard deviations from triplicates of each treatment.

Similarly, host specificity analysis revealed that phage KYP lysed only its susceptible bacteria *K. pneumoniae* KY and had no apparent impact on the other commensal bacteria isolated from the intestine of housefly larvae, including *K. pneumoniae* KX, *P. sneebia DSM* 19967, *P. faecalis*, *M. morganii*, *E. hormaechei*, *E. casseliflavus*, *P. stuartii* and *E. faecalis* (**Table S1**). It was observed by transmission electron microscopy that the head of phage KYP had a typical icosahedral structure and a short tail, which was in line with the short-tailed phage family (*Podoviridae*) (**Figure 2D**). The latent period of phage KYP was approximately 20 min, and then the titer increased sharply and reached the highest value, with a burst size of 3.28 × 10^12^/4.08 × 10^8^ = 8.04 × 10^3^ particles/infected cell (**Figure 4B**). When the MOI was 10^–2^, the phage KYP titer reached the highest level, approximately 12.61 log PFU mL-1, indicating that the OMOI of phage KYP was 10^-2^ (**Figure 4A**). It withstood a pH 5-11 environment for 1 h (**Figure 4C**). In addition, it withstood temperatures from 4°C to 60°C for 1 h (**Figure 4D**). In summary, the stability of phage KYP is good. Genome sequencing analysis showed that the total length of the KYP genome was 43,195 bp, and the average GC content was 55.99%, the number of protein-coding genes is 48, the gene length/genome ratio is 0.919 (**Figure S1B**, **Table S3**).

### Effects of *K. pneumoniae* KX/KY on the growth and development of housefly larvae

**Figure 5:**
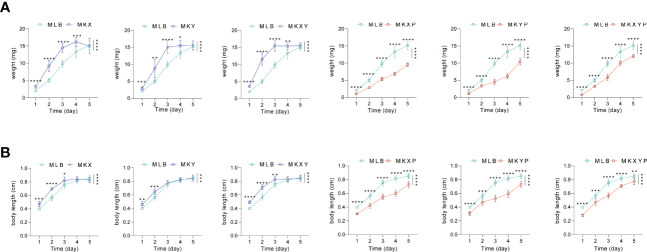
Effects of different treatments on the growth and development of housefly larvae. **(A)** Different treatments had significant effects on the body weight of housefly larvae. **(B)** Different treatments had significant effects on the body length of housefly larvae. MLB, MKX, MKY, MKXY, MKXP, MKYP and MKXYP were cultured in LB liquid medium, and (*K*) *pneumoniae* KX (10^9^ CFU/mL), (*K*) *pneumoniae* KY (10^9^ CFU/mL), (*K*) *pneumoniae* KX (10^9^ CFU/mL) and (*K*) *pneumoniae* KY (10^9^ CFU/mL), (*K*) *pneumoniae* phage KXP (10^7^ PFU/mL), (*K*) *pneumoniae* phage KYP (10^7^ PFU/mL), (*K*) *pneumoniae* phage KXP (10^7^ PFU/mL) and (*K*) *pneumoniae* phage KYP (10^7^ PFU/mL) were fed to housefly larvae. Each treatment included twelve biological repeats. The data are expressed as the means ± SEMs. Repeated measures ANOVA followed by Sidak correction was used for multiple comparisons. *p < 0.05, **p < 0.01, ***p < 0.001, ****P < 0.0001.

In order to analyze the effects of *K. pneumoniae* KX/KY and KX-KY mixture on larval development, feeding experiments were carried out. Based on our results, single feeding of *K. pneumoniae* KX (10^9^ colony forming unit (CFU)/mL), *K. pneumoniae* KY (10^9^ CFU/mL), and mixed feeding of *K. pneumoniae* KX (10^9^ CFU/mL) and *K. pneumoniae* KY (10^9^ CFU/mL) significantly promoted the growth and development of housefly larvae, but there was no synergistic effect on the growth and development of housefly larvae fed with mixed *K. pneumoniae* KX and KY. Feeding phage KXP (10^7^ PFU/mL), phage KYP (10^7^ PFU/mL), and mixed feeding of phages KXP (10^7^ PFU/mL) and KYP (10^7^ PFU/mL) significantly inhibited the growth and development of housefly larvae, but mixed feeding of phages KXP and KYP had no synergistic effect (*F* (24, 308) = 102.7, *P*<0.0001)(**Figure 5**). These results showed that compared with the control group, addition of *K. pneumoniae* KX/KY had a positive impact on the growth and development of housefly larvae, and the phage-mediated decrease in *K. pneumoniae* KX/KY had a negative impact on the growth and development of housefly larvae, thereby compromising larval health.

### Effects of the addition and removal of *K. pneumoniae* on intestinal microflora of housefly larvae

**Figure 6:**
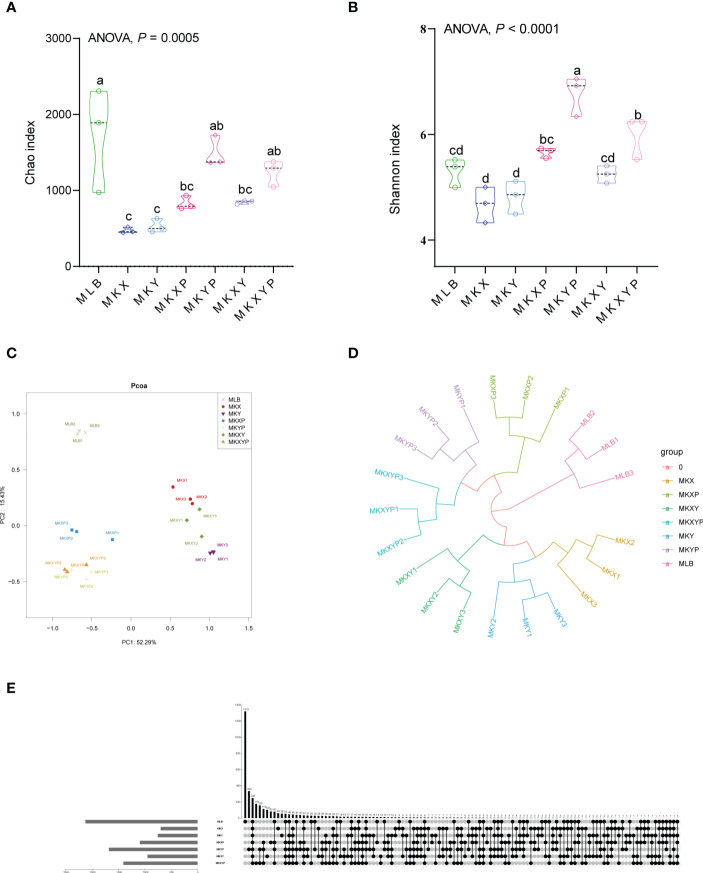
Bacterial richness and diversity of samples. **(A)** Chao1 index. **(B)** Shannon index. The data were compared by one-way ANOVA. The Brown-Forsythe test was used for significance analysis. Values are the means ± standard deviations from triplicates of each treatment. **(C)** Principal coordinate analysis (PCoA) of bacterial community structure in seven groups. Each symbol represents a sample of intestinal bacteria. **(D)** UPGMA tree analysis of the samples. **(E)** Venn diagram analysis of unique and shared OTUs of the intestinal bacteria in housefly larval samples. The number represents the number of unique OTUs in each sample and common OTUs shared by two or more samples.

To study the effect of the addition or lack of bacteria on the composition of the bacterial community, the 16S rRNA gene of intestinal bacteria of housefly larvae in group MLB, MKX, MKY, MKXY, MKXP, MKYP and MKXYP was sequenced. MLB, MKX, MKY, MKXY, MKXP, MKYP and MKXYP were cultured in LB liquid medium, *K. pneumoniae* KX (10^9^ CFU/mL), *K. pneumoniae* KY (10^9^ CFU/mL), *K. pneumoniae* KX (10^9^ CFU/mL) and *K. pneumoniae* KY (10^9^ CFU/mL), *K. pneumoniae* phage KXP (10^7^ PFU/mL), *K. pneumoniae* phage KYP (10^7^ PFU/mL), *K. pneumoniae* phage KXP (10^7^ PFU/mL) and *K. pneumoniae* phage KYP (10^7^ PFU/mL) were fed to housefly larvae. A total of 2,235,406 high-quality bacterial sequences with sequence numbers from 100025 to 116160 were produced from all samples. These sequences were normalized and clustered into 3,768 OTUs at a 97% similarity level among all the samples (**Table 1**). Chao1 indices showed that compared with the control group, the bacterial richness of housefly larvae in the phage-treated group (MKXP, MKYP and MKXYP) and bacteria-treated group (MKX, MKY and MKXY) decreased (**Figure 6A**). Shannon indices suggested that compared with the control group, the bacterial diversity of housefly larvae increased in the phage-treated group (MKXP, MKYP and MKXYP) but decreased in the bacteria-treated groups (MKX, MKY) (**Figure 6B**). PCoA analysis showed that there were significant differences in the intestinal microflora of housefly larvae between the phage treatment groups and the bacterial treatment groups (**Figure 6C**). The phage treatment groups (MKXP, MKYP and MKXYP) and bacterial treatment groups (MKX, MKY and MKXY) clustered together, and neither of them clustered with the control group (MLB). An unweighted pair-group method with arithmetic mean (UPGMA) tree further proved the aggregation of phage-treated group (MKXP, MKYP and MKXYP) and bacteria-treated group (MKX, MKY and MKXY) samples (**Figure 6D**). Therefore, the addition or lack of *K. pneumoniae* (KX, KY) significantly interfered with the larval intestinal flora.

**Table 1:** Information about 16S rRNA gene analysis in this study.

Sample		Clean Reads	Normalize d reads	OTU number	Shannon	Simpson	Chao1	Coverage
**MLB**	**MLB_1**	110478	81906	1085.00 ± 173.73	5.30 ± 0.22	0.9359 ± 0.0113	1723.71 ± 557.24	0.9958 ± 0.0020
	**MLB_2**	106403	81906					
	**MLB_3**	109817	81906					
**MKX**	**MKX_1**	106608	81906	432.67 ± 26.41	4.68 ± 0.27	0.9154 ± 0.0292	473.44 ± 32.67	0.9991 ± 0.0001
	**MKX_2**	105099	81906					
	**MKX_3**	103293	81906					
**MKY**	**MKY_1**	100543	81906	487.33 ± 67.34	4.82 ± 0.25	0.9260 ± 0.0137	528.17 ± 73.82	0.9992 ± 0.0001
	**MKY_2**	104974	81906					
	**MKY_3**	107099	81906					
**MKXY**	**MKXY_1**	100025	81906	634.67 ± 32.36	5.24 ± 0.13	0.9518 ± 0.0051	846.12 ± 20.19	0.9980 ± 0.0001
	**MKXY_2**	101407	81906					
	**MKXY_3**	116160	81906					
**MKXP**	**MKXP_1**	108512	81906	711.33 ± 18.93	5.66 ± 0.08	0.9452 ± 0.0022	829.28 ± 73.48	0.9983 ± 0.0004
	**MKXP_2**	104765	81906					
	**MKXP_3**	107643	81906					
**MKYP**	**MKYP_1**	109759	81906	1138.00 ± 96.44	6.77 ± 0.31	0.9773 ± 0.0065	1488.67 ± 169.12	0.9965 ± 0.0005
	**MKYP_2**	100510	81906					
	**MKYP_3**	109738	81906					
**MKXYP**	**MKXYP_1**	107371	81906	947.00 ± 72.20	6.00 ± 0.34	0.9538 ± 0.0172	1238.53 ± 139.80	0.9970 ± 0.0004
	**MKXYP_2**	107400	81906					
	**MKXYP_3**	107802	81906					

Data are expressed as the mean ± standard deviation of three replicate samples in each sampling.

The Venn diagram showed the common and unique OTUs of all samples, of which 248 OTUs (6.58%) were common to all samples (**Figure 6E**). There were 1319 (35.01%), 60 (1.59%), 56 (1.49%), 103 (2.73%), 82 (2.18%), 333 (8.84%) and 176 (4.67%) unique OTUs in the MLB, MKX, MKY, MKXY, MKXP, MKYP and MKXYP samples, respectively (**Figure 6E**). The Venn diagram showed the differences in the intestinal microflora of housefly larvae in different samples.

**Figure 7:**
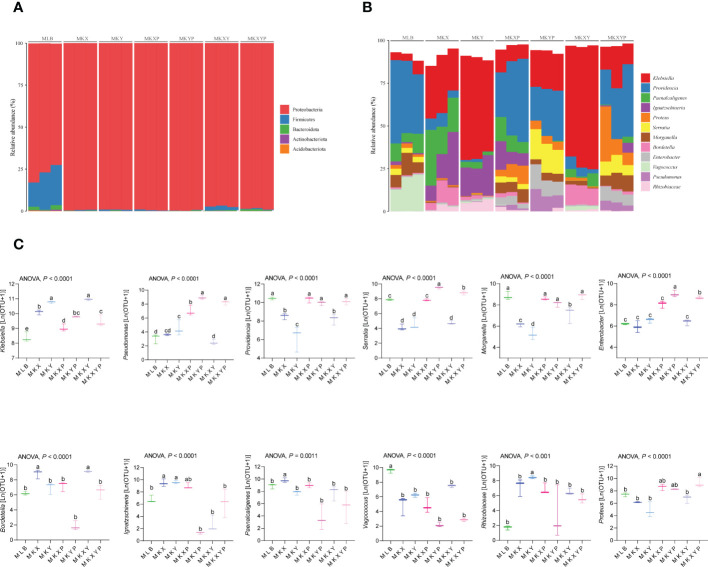
The relative abundances of the top 5 phyla and the top 12 genera of intestinal bacteria in different treatment groups. **(A)** Relative abundances of the top 5 phyla in housefly larval samples. **(B)** The relative abundances and distributions of the top 12 genera in housefly larval samples. **(C)** Dynamic variation in the OTU number of key bacteria in different groups. The data were compared by one-way ANOVA. The Brown-Forsythe test was used for significance analysis.

To further study the composition of the bacterial community in different groups, the composition and structure of the intestinal bacterial community were analyzed at different levels. Top 5 phyla and top 12 genera with relatively high abundance were selected for analysis. The results showed that the housefly larvae of different treatment groups had only slight differences at the phylum level, but had different bacterial community structures at the genus level. (**Figures 7A, B**). *Proteobacteria* accounted for the highest proportion of all intestinal bacteria in housefly larval samples (**Figure 7A**). In addition, in the bacterial treatment groups (MKX, MKY and MKXY) and phage treatment groups (MKXP, MKYP and MKXYP), the proportion of *Proteobacteria* increased, while *Firmicutes* and *Bacteroidota* decreased (**Figure 7A**). Among the 12 most abundant genera in the intestines of housefly larvae, the genus *Klebsiella* was significantly increased in abundance in the bacterial treatment group (MKX, MKY and MKXY), but the genus *Providencia*, *Serratia*, *Morganella*, and *Vagococcus* showed a significantly decreasing trend (**Figure 7B**). In the phage treatment group (MKXP, MKYP and MKXYP), the genus *Klebsiella* and *Pseudomonas* increased significantly in abundance, the genus *Proteus* increased slightly, and the genus *Vagococcus* decreased significantly (**Figure 7B**). The relative abundances of the genus *Serratia* and *Enterobacter* were significantly increased in the phage treatment group (MKYP, MKXYP) (**Figures 7B, C**). Therefore, the composition of the intestinal bacterial community of housefly larvae was greatly altered by the bacterial treatment group. Although phages have host specificity, they induce cascading effects on microbiota species that are not directly targeted. Similarly, the composition of the intestinal bacterial community of housefly larvae is greatly affected. Bacterial genera with great changes in abundance and proportion were investigated. In the group of larvae fed with *K. pneumoniae* KX/KY or with mixed KX and KY, the proportions of the genus *Klebsiella* and *Bordetella* were significantly increased while the abundance of the genus *Providencia*, *Serratia*, *Morganella* and *Vagococcus* decreased significantly. Significantly, the abundance of the genus *Paenalcaligenes* and *Enterobacter* showed the opposite trend in the two bacterial treatment groups (MKX, MKY). In the group of larvae fed with phage KXP/KYP or mixed KXP and KYP, the proportions of the genus *Klebsiella*, *Pseudomonas*, *Proteus* and *Enterobacter* were significantly increased, while the abundance of the genus *Providence* decreased slightly, and that of the genus *Vagococcus* decreased significantly. The proportion of the genus *Serratia* was significantly increased, while the abundance of the genus *Paenalcaligenes* was significantly decreased in the phage treatment group (MKYP, MKXYP). Similarly, the abundance of the genus *Paenalcaligenes*, *Ignatzschineria*, *Serratia* and *Bordetella* showed a reverse trend in the two phage treatment groups (MKXP, MKYP). Therefore, we speculate that in addition to the increase or phage-mediated decrease in *K. pneumoniae* (KX, KY), the change in the proportion of dominant bacteria, such as the genus *Klebsiella, Providence, Serratia, Morganella, Bordetella, Pseudomonas, Vagococcus* and *Enterobacter* in the intestinal tract of housefly larvae is another important factor affecting the health of houseflies. To determine the interaction between culturable bacteria and *K. pneumoniae* KX/KY, antagonism assays were carried out in aerobic cultures and facultative anaerobic cultures. The antagonism assay showed that the growth of *K. pneumoniae* KY, *P. aeruginosa* Y12 and *P. stuartii* Ps was inhibited by high abundances of *K. pneumoniae* KX, and the growth of *K. pneumoniae* KX, *P. aeruginosa* Y12 and *P. stuartii* Ps was also inhibited by high concentrations of *K. pneumoniae* KY (**Figures S2**, **3**).

### Network of gut microbiota in larvae treated with different bacteria amended diets

**Figure 8:**
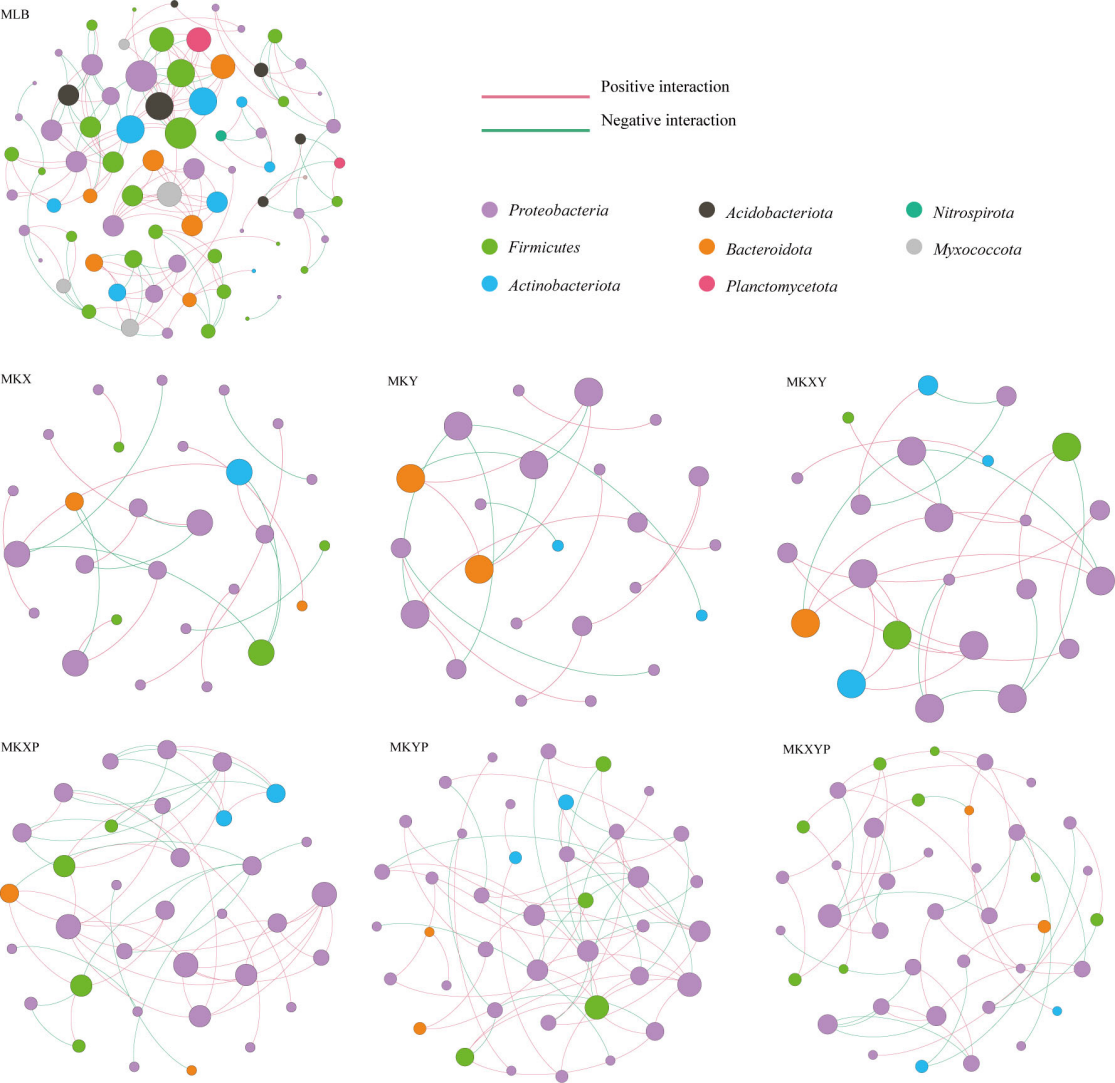
Intestinal bacterial cooccurrence microbiome networks between different processing groups. Each point in the graph represents a species, and those related species are connected by a line. The red lines represent positive correlations, and the green lines represent negative correlations. The node colors represent the taxon classification at the phylum level.

To analyse the bacterial correlations within the bacterial community, we constructed a network based on the Spearman correlation. Bacterial correlations among the larval intestinal flora were significantly changed after the larvae were treated with the two *K. pneumoniae* strains and *K. pneumoniae-*specific phages (**Figure 8**). Compared to the control group, oral administration of *K. pneumoniae* and phages significantly reduced bacterial correlations, especially in *Firmicutes* (except MKXYP), *Actinobacteriota* (except MKXY) and *Bacteroidota* (except MKY) (**Figure 8**). The total number of nodes, total links, average degree and average cluttering coefficient (except MKXY) of the interaction network in the gut microflora of the larvae decreased in the bacterial treatment group (MKX, MKY and MKXY) and phage treatment group (MKXP, MKYP and MKXYP) (**Table S1** **)**. We speculated that there was less contact between harmful bacteria in the bacterial treatment group and less contact between beneficial bacteria in the phage treatment group. However, compared with the bacterial treatment groups (MKX, MKY and MKXY), the phage-treated groups (MKXP, MKYP and MKXYP) had more total links and total nodes indicating that phage treatment increased the association between harmful bacteria (**Table S4** **)**. In all samples, a high degree of connectivity between *Proteobacteria* was observed (**Figure 8**). However, compared with the bacterial treatment group (47.62% in MKX, 65.00% in MKY and 42.31% in MKXY), the interaction between *Proteobacteria* increased in the phage-treated groups (59.26% in MKXP, 60.71% in MKYP and 67.44% in MKXYP). We speculate that there is an increased association between some harmful bacteria in *Proteobacteria* in phage-treated groups (MKXP, MKYP and MKXYP). Based on the results, alteration of the loads of specific bacteria in the intestine could greatly affect the bacterial interactions among the microbial communities.

### Bacterial assay of *K. pneumoniae* KX/KY in housefly larvae using specificity of their targeting phages under different conditions

**Figure 9:**
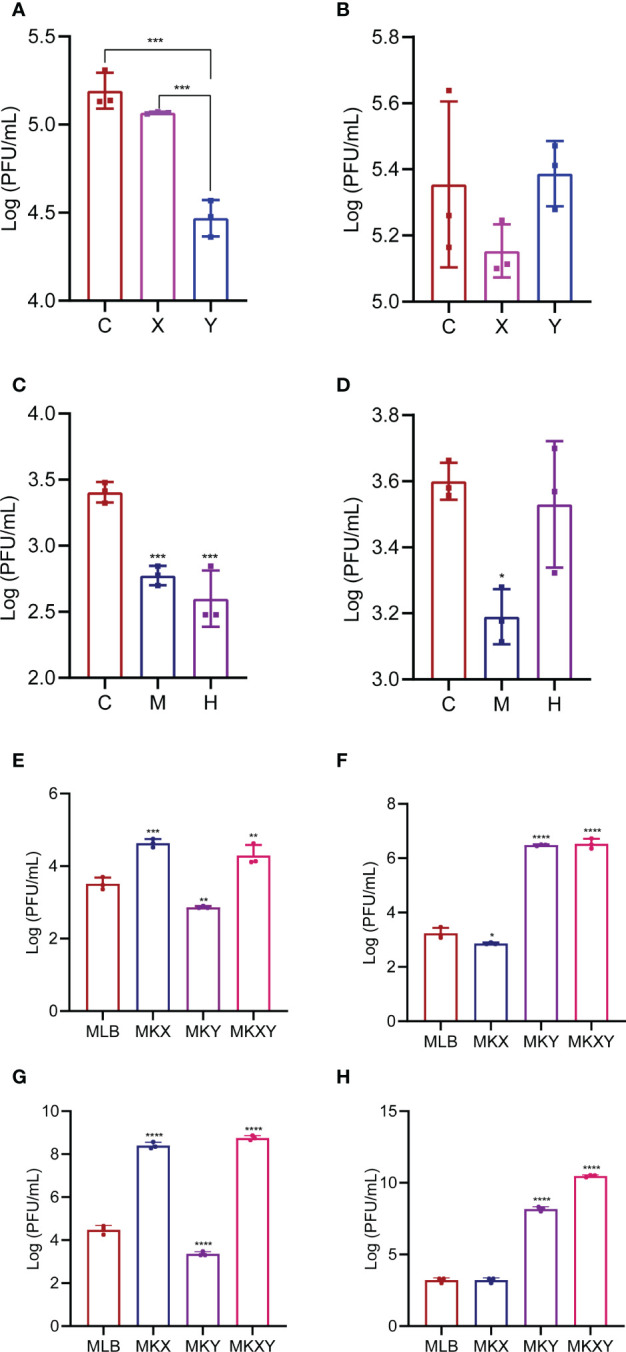
Bacterial assay of *Klebsiella pneumoniae* KX/KY under different conditions. **(A)** The titers of KXP in different groups under different oxygen concentrations, **(B)** The titers of KYP in different groups under different oxygen concentrations. C: control group, X: normal oxygen group, Y: low oxygen content culture group. **(C)** The titers of KXP in different treatment groups from the midgut and hindgut of housefly larvae, **(D)** The titers of KYP in different groups from the midgut and hindgut of housefly larvae. C: control group, M: the midgut group, H: the hindgut group. Values are the means ± standard deviations from triplicates of each treatment. **(E)** The titers of KXP in different groups feeding with various diets and placed on a shaking table for 1 hour, **(F)** The titers of KYP in different groups feeding with various diets and placed on a shaking table for 1 hour, **(G)** The titers of KXP in different groups feeding with various diets and placed on a shaking table for 4 hour, **(H)** The titers of KYP in different groups feeding with various diets and placed on a shaking table for 4 hour. MLB: feeding LB medium, MKX: feeding *Klebsiella pneumoniae* KX, MKY: feeding *Klebsiella pneumoniae* KY, MKXY: feeding *Klebsiella pneumoniae* KX-KY. Values are the means ± standard deviations from triplicates of each treatment. *p < 0.05, **p < 0.01, ***p < 0.001, ****P < 0.0001.

The dynamic analysis of *K. pneumoniae* KX/KY in housefly larvae under different oxygen concentrations revealed that phage KXP showed a higher titer in the environment under a high oxygen content while phage KYP revealed a higher titer in the environment under a low oxygen content. As phage KXP/KYP could rapidly amplify only in the presence of their host bacteria *K. pneumoniae* KX/KY, the titers of phages KXP/KYP revealed the result that a higher bacterial load of aerobic *K. pneumoniae* KX in the environment under a high oxygen content, while a higher bacterial load of anaerobic *K. pneumoniae* KY in the environment under a low oxygen content (**Figures 9A, B**). The results indicated that aerobic *K. pneumoniae* KX plays a major role in environments with a high oxygen content, while facultative anaerobic *K. pneumoniae* KY plays a major role in environments with a low oxygen content.

### Bacterial assay of *K. pneumoniae* KX/KY in the midgut and hindgut of housefly larvae

The midgut and hindgut maintain different oxygen concentration in housefly larvae. To analyze the dynamic alterations of *K. pneumoniae* KX/KY in the gut of housefly larvae with different oxygen concentrations, the bacterial load of *K. pneumoniae* KX/KY in the midgut and hindgut of housefly larvae was assayed. Based on the results, higher KXP titers was found in the midgut while higher KYP titers was found in hindgut indicating a higher bacterial load of aerobic *K. pneumoniae* KX in the high oxygen midgut while a higher bacterial load of anaerobic *K. pneumoniae* KY in the low oxygen hindgut. Our results revealed that aerobic *K. pneumoniae* KX plays a major role in the aerobic midgut, while facultative anaerobic *K. pneumoniae* KY plays a major role in the anaerobic hindgut (**Figures 9C, D**).

### Bacterial assay of *K. pneumoniae* KX/KY in housefly larvae after feeding *K. pneumoniae* KX/KY alone or feeding with mixed *K. pneumoniae* KX and KY

To further analyse the *in vivo* interactions between *K. pneumoniae* KX and KY in housefly larvae, the bacterial load of *K. pneumoniae* KX/KY in housefly larvae feeding *K. pneumoniae* KX/KY alone or mixed *K. pneumoniae* KX-KY was assayed through phage titer analysis. Phage titer analysis revealed that the phage KXP titer increased in the MKX and MKXY groups and decreased in the MKY group (**Figures 9E, G**). The phage KYP titer increased in groups MKY and MKXY, and the KYP titer decreased in group MKX (**Figures 9F, H**). The above experiments showed that the bacterial load of *K. pneumoniae* KX increased and that of KY decreased after the larvae were fed KX, while *K. pneumoniae* KY increased and KX decreased after the larvae were fed KY; both KX and KY increased after the larvae were fed mixed *K. pneumoniae* KX-KY. Therefore, we assume that *K. pneumoniae* KX and KY could maintain a dynamic balance in the housefly larvae and competitively facilitate larval development.

## Discussion

In this study, we isolated aerobic *K. pneumoniae* KX and facultative anaerobic *K. pneumoniae* KY, and phage KXP and phage KYP that specifically targeted the two strains of bacteria. The health of housefly larvae was positively affected by the increase in the abundance of *K. pneumoniae* KX/KY or two mixed bacteria and negatively affected by the decrease in the abundance of *K. pneumoniae* KX/KY or two mixed bacteria. Furthermore, the addition of *K. pneumoniae* KX/KY or phage KXP/KYP can affect the intestinal flora. Based on the results of our research, we speculate that the alteration of intestinal flora caused by the addition of *K.* pneumoniae KX/KY or phage KXP/KYP is an important factor affecting housefly larvae. These findings indicate the complexity of intestinal flora-host interactions in the intestinal tracts of insects and show that the phage predation not only knockdown their bacterial targets but also affect non-targeted species within a community of commensal bacteria colonizing the gut through cascading effects.

**Figure 10:**
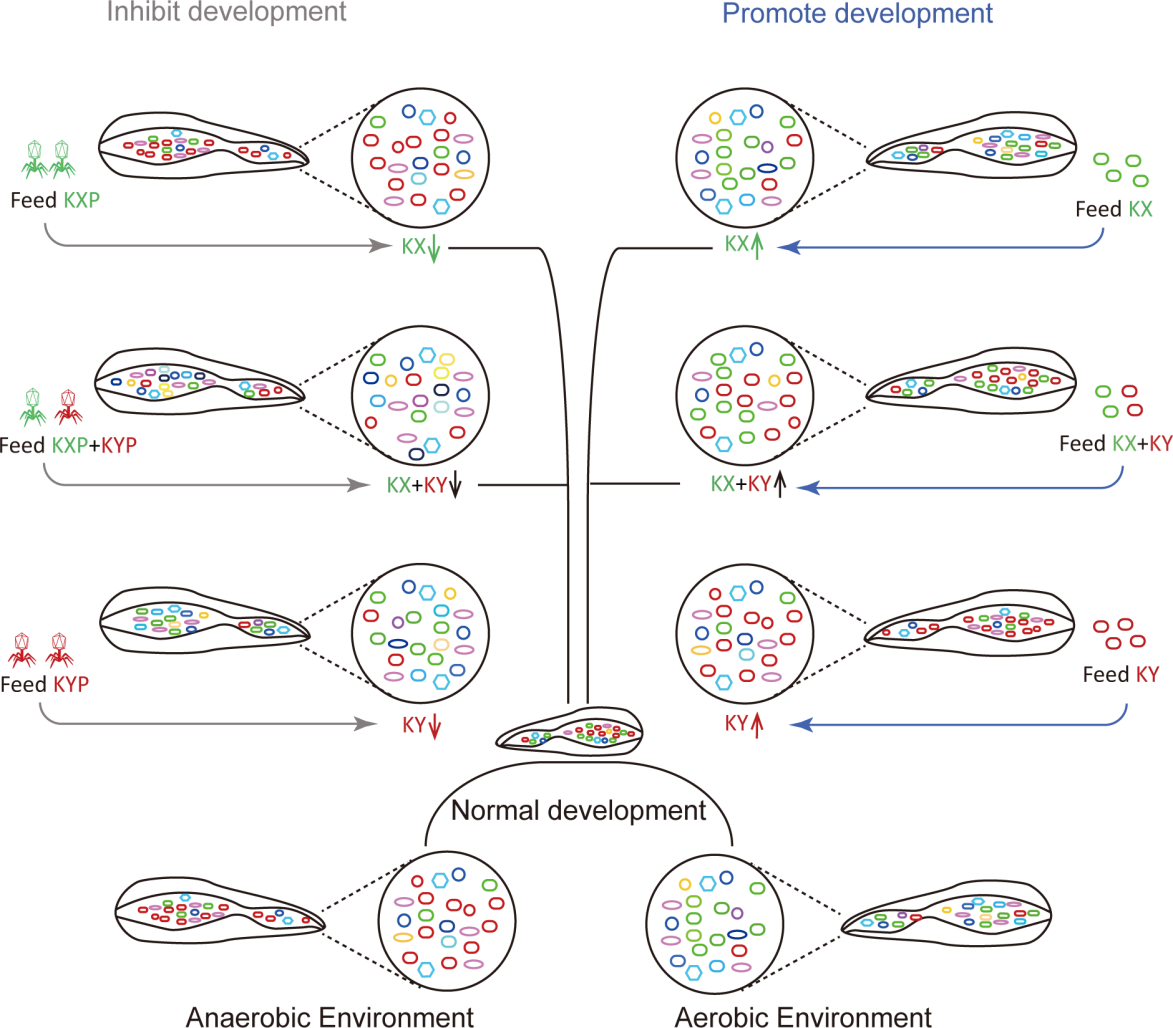
Patterns of the effects of two strains of *Klebsiella pneumoniae* on larval development of houseflies. Different colours represent different bacteria. Green, red and other colours represent *Klebsiella pneumoniae* KX, *Klebsiella pneumoniae* KY and other bacteria, respectively.

Pathogenic bacterial invasion can affect the growth of housefly larvae and even lead to their death (38). However, the effects of beneficial bacteria on the growth of housefly larvae need to be studied. In this study, we found that individual addition of *K. pneumoniae* KX/KY or the mixed addition of *K. pneumoniae* KX and KY in larval diets can affect the composition of intestinal microflora, inhibit the growth of harmful bacteria including *Pseudomonas aeruginosa* which inhibited housefly larval growth and accelerated larvae death at high bacterial concentration (38), and thus promote the development of housefly larvae (**Figure 10**). Using the *K. pneumoniae*-specific phage KXP/KYP to reduce the bacterial abundance of *K. pneumoniae* KX/KY in the larval gut microbiota, we found that the individual or mixed addition of phage KXP and KYP in larval diets can both affect the composition of intestinal microflora and promote the growth of harmful bacteria, thus inhibit the development of housefly larvae (**Figure 10**). Among the 12 genera most abundant in the intestinal flora of housefly larvae, *Klebsiella* was significantly increased in abundance in the bacteria-treated group (MKX, MKY and MKXY), while *Providencia*, *Serratia*, *Morganella*, and *Vagococcus* showed a significant decreasing trend. The abundance of *Klebsiella* in housefly larvae fed high concentrations of *K. pneumoniae* increased significantly in a short time, which could be attributed to the high concentrations of *K. pneumoniae* in the larval diet.

*Enterobacter* AA26 and *Klebsiella oxytoca* sp. have been used as probiotics in the diet of medfly. It also affected the feeding efficiency and biological quality of medfly VIENNA 8D53+ GSS (39). Given these effects of *Klebsiella*, it is assumed that *Klebsiella* will play an important role in the growth and development of housefly larvae. Among the bacteria that were observed to be reduced after larvae were fed *K. pneumoniae* KX/KY, *Providencia* (40, 41) and *Morganella* (42) species have been reported to be pathogenetic bacteria in insects, causing a high level of mortality in insects. *S. marcescens* digests the membrane-bound mucins on the intestinal epithelium of mosquitoes by secreting SmEnhancin protein, which destroys the intestinal physical barrier, thus enhancing the arbovirus infection in the intestinal epithelium of mosquitoes (11). Therefore, we assume that the elevated bacterial abundance of *K. pneumoniae* KX/KY caused a decrease in some harmful bacteria in the larval gut, which further favored the proliferation of other beneficial bacteria in the larval gut and benefited larval development.

To analyze the interaction between *K. pneumoniae* and other culturable bacteria, A plate antagonism experiment was carried out. We found that although *K. pneumoniae* KX and KY were different strains, they revealed the same inhibitory effects on the growth of *P. aeruginosa* and *P. stuartii.* Furthermore, they also showed inhibitory effects on each other. Based on the results of the plate antagonism experiment and gut community structure analysis, we assume that the elevated abundance of *K. pneumoniae* may result in it competing with other strains for nutrition and inhibit the growth of *P. stuartii* and *P. aeruginosa* in the intestinal tracts of housefly larvae, reducing the reproduction of harmful bacteria and promoting the growth and development of housefly larvae. The mutual inhibition between *K. pneumoniae* KX and *K. pneumoniae* KY explains why the mixed feeding of the two strains had no synergistic effect on the growth and development of housefly larvae. Furthermore, aerobic *K. pneumoniae* KX mainly exists in environments with a high oxygen content, while facultative anaerobic *K. pneumoniae* KY mainly exists in environments with a low oxygen content (**Figure 8**). Therefore, the two *K. pneumoniae* strains work together in the environment under different oxygen concentrations. Obviously, when these two bacteria in the intestines of housefly larvae increase to a certain extent, they will inhibit each other to achieve a certain balance. We speculate that the change in intestinal flora will protect larvae from pathogen invasion and promote the growth of host larvae.

Studies have shown that the biocontrol effect of phage combinations is improved by increasing the concentration of phages (43, 44). Bacterial resistance during phage therapy is restricted by the receptors or mechanisms of various phages (45, 46). In this experiment, two-phage combinations were used to feed housefly larvae to study whether the combined application of phages has a synergistic effect. Single phage KXP or KYP treatment had a significant inhibitory effect on the growth and development of housefly larvae, but the inhibitory effect did not increase when phages were combined applied, indicating that *K. pneumoniae* KX and KY co-existed and inhibited each other in the larval gut. After the phage KXP mediated a decrease in *K. pneumoniae* KX to a certain extent, *K. pneumoniae* KY tended to increase because the inhibition of *K. pneumoniae* KX on *K. pneumoniae* KY was weakened. The increasing trend of *K. pneumoniae* KY was opposite to the decreasing trend of *K. pneumoniae* KY mediated by phage KYP. When *K. pneumoniae* KX decreased, *K. pneumoniae* KY increased relatively, and the two bacterial population levels reached a certain balance.

The function and composition of insect intestinal flora are dynamic, and the interbacterial interactions is crucial to the health of insects. Therefore, we studied the structure of the intestinal microflora after an increase or phage-mediated decrease in *K. pneumoniae* in housefly larvae and analysed the interaction between them. The results showed that the structure of the intestinal flora changed after the increase or phage-mediated decrease in *K. pneumoniae*, and there were significant differences in network interactions among different strains. Compared with the control group MLB, the bacterial treatment groups MKX, MKY and MKXY and the phage treatment groups MKXP, MKYP and MKXYP increased the negative correlations among flora, indicating intensified competition among bacteria. When *K. pneumoniae* is added, *K. pneumoniae* will compete with some bacteria for resources, resulting in a decrease in harmful bacteria such as *Providence* and *Serratia.* A phage-mediated decrease in *K. pneumoniae* will induce an increase in harmful bacteria. These increased bacteria, such as *Serratia* and *Pseudomonas*, will also compete with each other and then affect the health of housefly larvae. In addition, the network diagram showed that eight phyla, *Proteobacteria*, *Firmicutes*, *Actinobacteriota*, *Acidobacteriota*, *Bacteroidota*, *Planctomycetota, Nitrospirota* and *Myxococcota*, were found in the control group. However, there were only *Proteobacteria*, *Firmicutes* (except MKY), *Actinobacteriota* and *Bacteroidota* in the bacterial treatment group (MKX, MKY and MKXY) and phage treatment group (MKXP, MKYP and MKXYP). Compared with the control group, the connection between the flora in the bacterial treatment groups MKX, MKY and MKXY and the phage treatment groups MKXP, MKYP and MKXYP was weakened. We speculated that the addition of *K. pneumoniae* KX/KY to larval diets reduced the contact between harmful bacteria and that the addition of the *K. pneumoniae*-specific phage KXP/KYP reduced the contact between beneficial bacteria. Compared with the bacteria-treated groups MKX, MKY and MKXY, the flora were more closely related, and the interaction between *Proteobacteria* increased in the phage-treated groups MKXP, MKYP and MKXYP. We speculated that the association between harmful bacteria in *Proteobacteria*, such as *Serratia* and *Pseudomonas*, increased in the phage-treated groups MKXP, MKYP and MKXYP. Therefore, an increase or phage-mediated decrease in *K. pneumoniae* in the intestine greatly affects the interaction between other bacterial groups.

According to our results, we believe that the factors contributing to the rapid growth of larvae caused by the addition of single *K. pneumoniae* KX/KY or *K. pneumoniae* KX and KY mixtures are as follows: (i) A large number of *K. pneumoniae* consume a large amount of nutrients or produce substances that inhibit the growth of pathogens to inhibit the growth of intestinal pathogens. (ii) The increase in *K. pneumoniae* caused significant changes in the intestinal flora of housefly larvae, resulting in complex interactions between the flora. There are two main factors that can explain why phage treatment inhibits the development of housefly larvae. The first reason is that the phage-mediated decrease in *K. pneumoniae* KX/KY will provide more nutritional resources and space for other harmful bacteria. The second reason is that reduction in *K. pneumoniae* abundance disturbed the balance of intestinal flora, which further affected the development of housefly larvae. We believe that the interaction between phages and their host bacteria affects the growth and development of insects by changing the relationship between bacteria and insects. This ecological evolutionary feedback is widely found in various systems. Our study analysed the interaction between phages, intestinal bacteria and insects in the intestinal environment and revealed the importance of gut bacteria in larval development.

In conclusion, this study investigated the dynamic changes in intestinal microflora caused by aerobic and facultative anaerobic *K. pneumoniae* strains using phages as a targeting tool. Based on our research, aerobic *K. pneumoniae* KX and facultative anaerobic *K. pneumoniae* KY plays necessary role in the aerobic midgut and anaerobic hindgut of housefly, respectively. Our work provides a framework to guide these future investigations in more complex environments that will seek to elucidate the interplay between insect aerobic/anaerobic microbes and insect health.

## Methods

### Animal and microbial strains

A housefly (*Musca domestica*) colony was reared and maintained in the Laboratory of Vector and Insect Diseases of Shandong First Medical University since 2005 (36).

Using traditional isolation and culture methods and anaerobic techniques (anaerobic airbag and anaerobic tank), *K. pneumoniae* KX was isolated from the intestine of 3-day-old housefly larvae under aerobic condition, and *K. pneumoniae* KY was isolated from the intestine of 3-day-old housefly larvae under facultative anaerobic condition (7). The isolated strains were sent to Qingdao sequencing Department of Beijing Ruibo Xingke Biotechnology Co., Ltd. for sequencing. The 16s rDNA was amplified with the primers 27F (AGAGTTTGATCCTGGCTCAG) and 1492R (TACGGCTACCTTGTTACGACTT). The sequence was blast in the GenBank database to search for the most similar sequences. The retrieved homologous sequences were analyzed with MEGA11.0 software to construct phylogenetic tree by ML bayesian method.

### Bacteriophage isolation and identification

In this experiment, sewage from Taian treatment plant in Shandong Province was collected for phage isolation. *K. pneumoniae* KX isolated from the intestinal tract of housefly larvae under aerobic condition and *K. pneumoniae* KY isolated from facultative anaerobic state were used as host bacteria to screen phages. Phage KXP were purified by a double-layer agar plate method as reported in our previous research (36). Except that the culture state is facultative anaerobic, the process of phage KYP separation is the same as that of phage KXP. The high concentration phage stocks were stored at 4°C. The purified phage preparation was imaged under transmission electron microscope (H-7700, Hitachi, Japan).

In order to confirm the host specificity (47, 48) of phage KXP, the spot titer technique was used. Take 10 μL 10^8^ PFU/mL phage KXP and drop it on the double-layer agar plate overlays of each bacteria (without phage) including *P. sneebia DSM* 19967, *P. faecalis, M. morganii*,*E. hormaechei, E. casseliflavus, P. stuartii, E. faecalis, K. pneumoniae* KX, *K. pneumoniae* KY were isolated from the intestine of housefly larvae and plates were incubated overnight at 37°C aerobically depending on the bacterial culture conditions. Under the condition of co-culture with *K. pneumoniae* KX, the OMOI of phage KXP was determined (49). Under aerobic condition, *K. pneumoniae* KX was cultured in LB liquid medium (yeast extract 5.0 g liter^-1^, tryptone 10.0 g liter^-1^, NaCl 10.0 g liter^-1^) to the logarithmic growth phase (OD 600 = 0.6). Phage KXP and host *K. pneumoniae* KX (10^8^ CFU/mL) were co-cultured at different MOIs ((10, 1, 10^-2^, 10^-4^, 10^-6^)). After incubated at 37°C for 3.5h, the phage KXP titer of supernatant was determined. The group with the highest titer is the OMOI of the phage KXP. The one-step growth curve experiment was carried out using the method reported in our previous research (37). One hundred microliter of phage KXP was mixed with 900 µL of SM buffer in different pH values (4–12), then incubated in a water bathe at 37°C. Samples were taken out after 3.5 h, and the potency was measured. One milliliter of phage KXP was incubated in a water bath at 4°C, 25°C, 37°C, 50°C, 60°C and 70°C, respectively. Samples were taken out after 1h to measure the titers. The titer of phage was determined by double-layer agar plate method. All analyses were performed as previously described, with some modifications, and were performed in triplicate. Except that the culture state is facultative anaerobic, the biological determination method of phage KYP was the same as that of phage KXP.

### Phage genome sequencing and bioinformatics analysis

Phage chromosomal DNA was isolated using the λ phage genomic DNA purification kit (ABigen) following the manufacturer’s instructions. Whole-genome sequencing was performed with an Illumina HiSeq 4000 platform. The METAVIRALSPADES pipeline (50) was used to identify the phage in the sample (mainly including sequence assembly, phage sequence identification, and phage integrity identification). The whole gene sequence was predicted by GeneMarkS software, the tRNA gene in the whole genome was predicted by tRNAscan-SE, the rRNA gene was predicted by Barrnap, and the prediction of other non-coding RNA was mainly obtained by comparing with Rfam database. The functional annotation of protein coding genes was compared with Swiss-Prot, NR, pfam, GO, KEGG and other databases by blastp. Annotated genome map was made using the criclize package in R. Phage sequences were deposited in the NCBI (KXP: ON755175, KYP: ON755176).

### Experimental design of insect feeding and *K. pneumoniae* (KX and KY) and phage (KXP and KYP)

To study the effects of *K. pneumoniae* KX/KY on the growth and development of housefly larvae. *K. pneumoniae* KX/KY was inoculated in freshly prepared LB liquid medium and placed in a constant temperature shaker. The concentration of *K. pneumoniae* KX/KY was about 109 CFU/mL after shaking at 110 rpm at 37°C for 24 h, which could be used as a high concentration of *K. pneumoniae*. To study the effects of *K. pneumoniae* KX-KY mixture on the growth and development of housefly larvae, *K. pneumoniae* KX and KY were mixed at 1:1 at the same concentration of 10^9^ CFU/mL. Effects of *K. pneumoniae* phage KXP on the growth and development of housefiy larvae. The feeding experiment of phage KXP was designed as follows: polyethylene glycol (PEG) precipitation was used to enrich the phage particles, and the high concentration of phage KXP was about 10^11^ PFU/mL. Phage KXP was diluted into 10^7^ PFU/mL with LB liquid medium. The feeding experiment on the effects of *K. pneumoniae* phage KYP on the growth and development of housefly larvae was the same as above. Housefly larvae from different groups that were fed LB liquid medium, *K. pneumoniae* KX (10^9^ CFU/mL), *K. pneumoniae* KY (10^9^ CFU/mL), *K. pneumoniae* KX (10^9^ CFU/mL) and *K. pneumoniae* KY (10^9^ CFU/mL), *K. pneumoniae* phage KXP (10^7^ PFU/mL), *K. pneumoniae* phage KYP (10^7^ PFU/mL), *K. pneumoniae* phage KXP (10^7^ PFU/mL) and *K. pneumoniae* phage KYP (10^7^ PFU/mL) were named MLB, MKX, MKY, MKXY, MKXP, MKYP and MKXYP respectively. The solutions of different treated groups were mixed with sterilized wheat bran at 1:1 ratio, respectively, and placed in a 5 mL perforated tube to ensure ventilation. The same amount of wheat bran (1.5 g~1.6 g) was placed in each tube, and then 10 normal-breeding, good-growing, uniform-sized 1-day-old larvae were added to each centrifuge tube. Each group is repeated three times and a piece of gauze is placed between the tube and the lid to prevent the larvae from escaping. They were placed in an artificial climate chest at 25 ± 1°C, a 50-55% relative humidity (RH), and a photoperiod of LD 12:12 h. Each group of experiments were carried out in three perforated test tubes, and four samples were taken from each tube every day as repetition. The body length and weight of housefly larvae were recorded regularly every day.

The surface debris of housefly larvae was removed and placed in a 1.5 mL centrifuge tube containing 75% alcohol, soaked and disinfected 10min, and then rinsed with sterile deionized water to remove bacteria adhering to the surface. After strict body surface disinfection, the housefly larva samples were stored in -80°C, and the samples of housefly larval on the day with the largest difference were selected for 16S rRNA high-throughput sequencing. Five larvae taken from different treatment groups and control groups were taken as sampling units, and there were 3 repeats in each group (38).

### Plate confrontation assay between *K. pneumoniae* and cultivable bacteria isolated from the gut of houseflies

In order to determine the interaction between culturable bacteria and *K. pneumoniae* (KX and KY) and between *K. pneumoniae* KX and *K. pneumoniae* KY of housefly larvae, plate confrontation assay were carried out in nutrient agar (NA) medium plates (peptone 10.0 g liter^-1^, agar 20 g liter^-1^, NaCl 5.0 g liter^-1^, beef extract 3.0 g liter^-1^) under aerobic and facultative anaerobic culture conditions. *K. pneumoniae* KX, *K. pneumoniae* KY, *P. aeruginosa* Y12 and *Providencia stuartii* Ps were inoculated in LB liquid medium (yeast extract 5.0 g liter^-1^, tryptone 10.0 g liter^-1^, NaCl 10.0 g liter^-1^) and cultured at 37°C overnight (OD600 >1.0). *K. pneumoniae* KX cultures was inoculated on half of the nutrient agar plate using the spread plate method with a sterile cotton swab, and the opposite side of the agar plate was used as a negative control. Two 6mm-diameter sterile filter papers were placed symmetrically on both sides of the agar medium. 10 µL of the isolated cultivable gut bacteria, including *K. pneumoniae* KY, *P. aeruginosa* Y12 and *P. stuartii* Ps were added to the filter papers. Similarly, *K. pneumoniae* KY cultures was inoculated on half of the nutrient agar plate using the spread plate method with a sterile cotton swab, and the opposite side of the agar plate was used as a negative control. Two 6mm-diameter sterile filter papers were placed symmetrically on both sides of the agar medium. 10 µL of the isolated cultivable gut bacteria, including *K. pneumoniae* KX, *P. aeruginosa* Y12 and *P. stuartii* Ps were added to the filter papers. All plates were cultured at 37°C and cultured in aerobic and anaerobic conditions for 48 h. Finally, the growth diameter of bacteria was recorded. The interaction between them was evaluated by measuring the colony size of *K. pneumoniae* KX, *K. pneumoniae* KY, *P. aeruginosa* Y12 and *P. stuartii* Ps. The experiments were conducted with six independent biological replications.

### Sequencing and bioinformatics analysis

DNA extraction of the intestinal bacteria and determining changes in gut composition using Illumina MiSeq Sequencing were carried out using the same method as described in our previous research (36). MENAP is used to analyze the OTU data of each sample, and the edge and node data are obtained. The network was performed using Gephi. The parameters used in drawing mainly include: node size is degree (degree: the degree of connection, the more connections, the higher the degree), the node color is phylum classification, the edge color is positive and negative correlation, and the node layout is Fruchterman Reingold.

### Bacterial assay of *Klebsiella pneumoniae* KX/KY in housefly larvae grown under different oxygen concentrations

Based on the highly specificity of phages, phage KXP/KYP could rapidly amplify only in the presence of their host bacteria. Therefore, bacterial load of *K. pneumoniae* KX/KY in housefly could be analyzed by measuring the titers of their specific phages *in vitro*. In order to analyze the load of *K. pneumoniae* KX and *K. pneumoniae* KY in housefly larvae grown under different oxygen concentrations, phage titer analysis was carried out. In the normal oxygen content culture group, LB liquid medium was mixed with sterilized wheat bran at a 1:1 ratio while the ration of LB liquid medium was increased (1.5:1) to create the low oxygen environment. The same amount of wheat bran (1.5 g~1.6 g) was placed in each tube, and then 10 uniform-sized 1-day-old larvae with normal breeding behavior and good growth were added to each centrifuge tube. Each group was repeated three times, and a piece of gauze was placed between the tube and the lid to prevent the larvae from escaping. Larvae were placed in an artificial climate chest at 25 ± 1°C, 50-55% relative humidity (RH), and a photoperiod of LD 12:12 h. After the larvae were 3 days old, the larvae cultured in normal oxygen and low oxygen environment were collected and ground thoroughly to obtain a uniform grinding solution as the stock solution. The larvae cultured in the normal oxygen group were taken as an example. The larvae were removed from the 5 mL centrifuge tube and placed in a 1.5 mL centrifuge tube. The larvae were killed by being placed in the freezer at -20°C for a few minutes. Then, the larvae were removed, soaked in alcohol for 2 min, and rinsed with sterile water three times to thoroughly disinfect their body surface. PBS (1000 μL) was added to a 1.5 mL centrifuge tube, ground thoroughly with a grinding rod after high-pressure sterilization, and shaken with a shaker for 1 min to obtain a uniform grinding solution as the stock solution. Then, PBS buffer was used to dilute the uniform grinding solution to obtain a 10^-4^ grinding solution. The treatment of larvae in the low oxygen content culture group was the same as above. Each group had 3 parallels. Then, 3 groups were established. Control group: 0.1 mL KXP (10^7^ PFU/mL) and 9.9 mL LB were mixed, and 0.1 mL KYP (10^7^ PFU/mL) and 9.9 mL LB were mixed. Normal oxygen content culture group: after mixing 1 mL grinding solution (10^–4^) and 20 mL LB, the bacteria were shaken on a shaking table for 12 h to obtain bacterial solution X1. Then, 1 mL bacterial solution X1, 0.1 mL KXP (10^7^ PFU/mL) and 8.9 mL LB were mixed, and 1 mL bacterial solution X1, 0.1 mL KYP (10^7^ PFU/mL) and 8.9 mL LB were mixed. Low oxygen content culture group: The experimental process was the same as above. Each group was analyzed in triplicate. The mixed solutions of different treatment groups were placed on a shaking table for 2 h and then filtered to measure the titers of KXP and KYP in different groups to determine the load of *K. pneumoniae* KX and KY in the housefly larvae under the two culture conditions.

### Bacterial assay of *Klebsiella pneumoniae* KX/KY in the midgut and hindgut of housefly larvae

As the midgut and hindgut contain different oxygen conditions, phage titers analysis was incorporated to analyze the dynamic bacterial load of *K. pneumoniae* KX and *K. pneumoniae* KY in midgut and hindgut.

The normally reared housefly larvae were cultured to 3-day-old larvae, and the midgut and hindgut were taken with a scalpe in clean bench for further analysis. The specific operations are as follows. First, the 3-day-old housefly larvae were taken out and placed in a 1.5 mL centrifuge tube. The larvae were killed by placing them in the refrigerator at -20°C for a few minute. Then, the larvae were removed, soaked in alcohol for 2 min, and rinsed with sterile water three times to thoroughly disinfect their body surface. Then the housefly larvae were dissected and the midgut and hindgut were taken in clean bench.

Take 6 portions of midgut and put it in 1000 μL of PBS in a 1.5 mL centrifuge tube, ground thoroughly with a sterile grinding rod, and shaken with a shaker for 1 min to obtain a uniform grinding solution as the stock solution. Then, PBS buffer was used to dilute the uniform grinding solution to obtain a 10^-4^ grinding solution. The treatment of hindgut group of housefly larvae was the same as above. Each group had 3 parallels. Then, 3 groups were established. Control group: 0.1 mL KXP (10^5^ PFU/mL) and 9.9 mL LB were mixed, and 0.1 mL KYP (10^5^ PFU/mL) and 9.9 mL LB were mixed. Midgut group: after mixing 1 mL of 10^-4^ grinding solution and 20 mL LB, the bacteria were shaken on a shaking table for 12 h to obtain bacterial solution M1. Then, 1 mL bacterial solution M1, 0.1 mL KXP (10^5^ PFU/mL) and 8.9 mL LB were mixed, and 1 mL bacterial solution M1, 0.1 mL KYP (10^5^ PFU/mL) and 8.9 mL LB were mixed. Hindgut group: The experimental process was the same as above. Each group was analyzed in triplicate. The mixed solutions of different treated groups were placed on a shaking table for 4 h and then filtered to measure the titers of KXP and KYP in different groups to determine the concentrations of *K. pneumoniae* KX and KY in the midgut and hindgut of housefly larvae.

### Bacterial assay of *Klebsiella pneumoniae* KX/KY in housefly larvae after feeding *Klebsiella pneumoniae* KX/KY alone or feeding with mixed *Klebsiella pneumoniae* KX and KY

The 1-day-old larvae in the control group were fed LB medium (group MLB), while others were fed *K. pneumoniae* KX (group MKX, 10^9^ CFU/mL), *K. pneumoniae* KY (group MKY, 10^9^ CFU/mL) and *K. pneumoniae* KX-KY (group MKXY, 10^9^ CFU/mL) until they grew to 3-day-old larvae. Phages KXP and KYP were used to measure the concentrations of *K. pneumoniae* KX and *K. pneumoniae* KY in housefly larvae in the four groups. In the control group, LB liquid medium was mixed with sterilized wheat bran at a 1:1 ratio and placed in a 5 mL perforated tube to ensure ventilation. In the MKX group, *K. pneumoniae* KX (10^9^ CFU/mL) bacterial solution was mixed with sterilized wheat bran at a 1:1 ratio and placed in a 5 mL perforated tube to ensure ventilation. In the MKY group, *K. pneumoniae* KY (10^9^ CFU/mL) bacterial solution was mixed with sterilized wheat bran at a 1:1 ratio and placed in a 5 mL perforated tube to ensure ventilation. In the MKXY group, both *K. pneumoniae* KX (109 CFU/mL) and *K. pneumoniae* KY (109 CFU/mL) were mixed with sterilized wheat bran at a 1:1 ratio and placed in a 5 mL perforated tube to ensure ventilation. Each treatment was repeated three times, and a piece of gauze was placed between the tube and the lid to prevent the larvae from escaping. Larvae were placed in an artificial climate chest at 25 ± 1 °C, 50-55% relative humidity (RH), and a photoperiod of LD 12:12 h. After three days, the 3-day-old larvae were removed from the 5 mL centrifuge tube and placed in a 1.5 mL centrifuge tube. The larvae were killed by placing them in the refrigerator at -20°C for a few minutes. Then, the larvae were removed, soaked in alcohol for 2 min, and rinsed with sterile water three times to thoroughly disinfect their body surface. PBS (1000 μL) was added to a 1.5 mL centrifuge tube, ground thoroughly with a grinding rod after high-pressure sterilization, and shaken for 1 min to obtain a uniform grinding solution as the stock solution. The stock solution was 10^-4^ diluted with PBS buffer. Then, 1 mL of the 10^-4^ diluted solution was transferred to a sterile conical flask mixed with 20 mL of LB, and the conical flasks were cultured for 12 h to obtain bacterial solution C1. Then, 1 mL of bacterial solution C1, 0.1 mL of KXP (10^5^ PFU/mL) and 8.9 mL of LB were mixed, and 1 mL of bacterial solution C1, 0.1 mL of KYP (10^5^ PFU/mL) and 8.9 mL of LB were mixed. After shaking for 1 and 4 h, the cultures were filtered to measure the titers of KXP and KYP to determine the concentrations of *K. pneumoniae* KX/KY in housefly larvae in different groups. The experimental processes for the MKX, MKY and MKXY groups were the same as above. Each group was analysed in triplicate.

## Statistical analysis

All data analysis was performed by IBM SPSS Statistics 20 statistical software. All data are expressed as mean ± SD. Two-way ANOVA was used to compare the changes of body weight and body length of housefly larvae under different treatments. Significance analysis was performed by Sidak’s multiple comparisons test (*p* < 0.05).

## Data availability statement

The datasets presented in this study can be found in online repositories. The names of the repository/repositories and accession number(s) can be found in the article/supplementary material.

## Author contributions

RZ and ZZ conceived and designed the experiments. SW and DY supervised the project. KZ, XZ, QZ, WL, YL, YY and SA performed the experiments. All authors analyzed and discussed the data. KZ, SW and DY wrote the manuscript. RZ and ZZ revised the manuscript. All authors contributed to the article and approved the submitted version.
