# Supplementary material for: Aerobic and facultative anaerobic Klebsiella pneumoniae strains establish mutual competition and jointly promote Musca domestica development
Source: Front Immunol. 2023 Feb 17;14:1102065. doi: 10.3389/fimmu.2023.1102065 (PMC9982019; doi:10.3389/fimmu.2023.1102065)
Supplement: Supplementary file 1 [file DataSheet_1.docx]

**Supplementary Material**

**Table S1.** Infectivity range of phages against intestinal bacteria of housefly larvae. Related to Figure 1. Phages were spotted onto lawns of each bacteria and incubated anaerobically overnight at 37 °C. Zones of clearing indicated infectivity. (+) = lysis; (-) = no lysis

| **Bacteria** | **Phage** | |
| --- | --- | --- |
|  | KXP | KYP |
| *Providencia sneebia DSM* 19967 | － | － |
| *Pseudocitrobacter faecalis* | － | － |
| *Morganella morganii* | － | － |
| *Enterobacter hormaechei* | － | － |
| *Enterococcus casseliflavus* | － | － |
| *P. stuartii* | － | － |
| *Enterococcus faecalis* | － | － |
| *K. pneumoniae*(KX) | ＋ | － |
| *K. pneumoniae*(KY) | － | ＋ |

**Supplementary Tables:**

**Table S4** The co-occurrence network indices of the different treatment groups.

| **Treatment group** | **Total nodes** | **Total links** | **Average degree** | **Average cluttering coefficient** | **Average path distance** | **Positive correlation** | **Negative correlation** |
| --- | --- | --- | --- | --- | --- | --- | --- |
| **MLB** | 78 | 158 | 4.051 | 0.793 | 1.73 | 67.09 | 32.91 |
| **MKX** | 27 | 21 | 1.556 | 0.733 | 1.31 | 52.38 | 47.62 |
| **MKY** | 23 | 20 | 1.739 | 0.515 | 1.576 | 60 | 40 |
| **MKXY** | 23 | 26 | 2.261 | 0.926 | 1.167 | 65.38 | 34.62 |
| **MKXP** | 34 | 54 | 3.176 | 0.78 | 2.135 | 64.81 | 35.19 |
| **MKYP** | 40 | 56 | 2.8 | 0.762 | 1.717 | 64.29 | 35.71 |
| **MKXYP** | 40 | 43 | 2.15 | 0.682 | 2.208 | 58.14 | 41.86 |

**Supplementary Figures:**

**
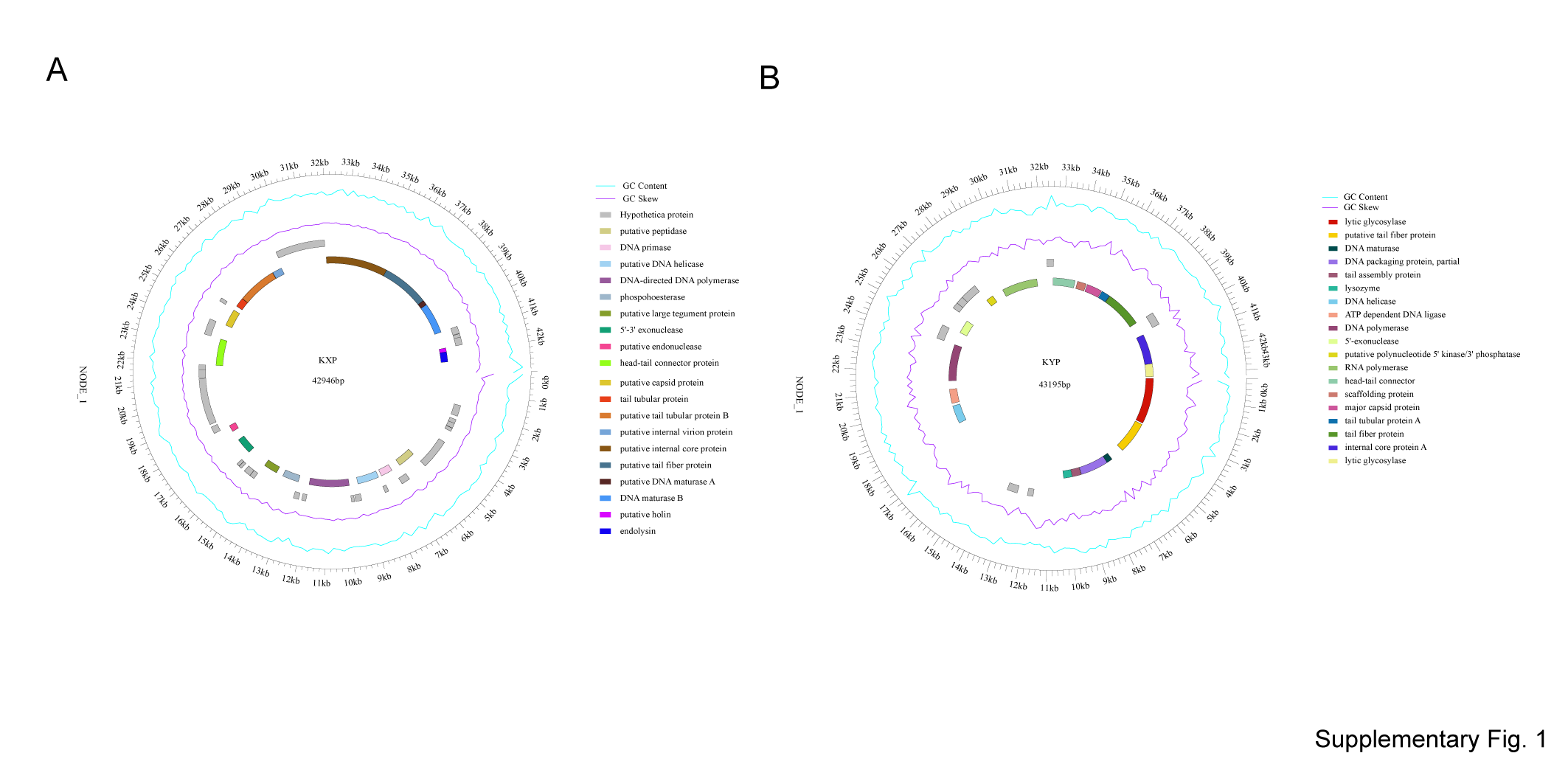
**

**Figure S1 | Annotated genome maps for the phage KXP (A) and phage KYP (B).** In the circular genome map, the outermost black circle represents the full length of the genome, the innermost multicoloured circle represents annotated functional proteins, the second outermost blue circle represents GC skew, the third outermost purple circle represents GC content, and the fourth outermost grey circle represents hypothetical proteins.

**
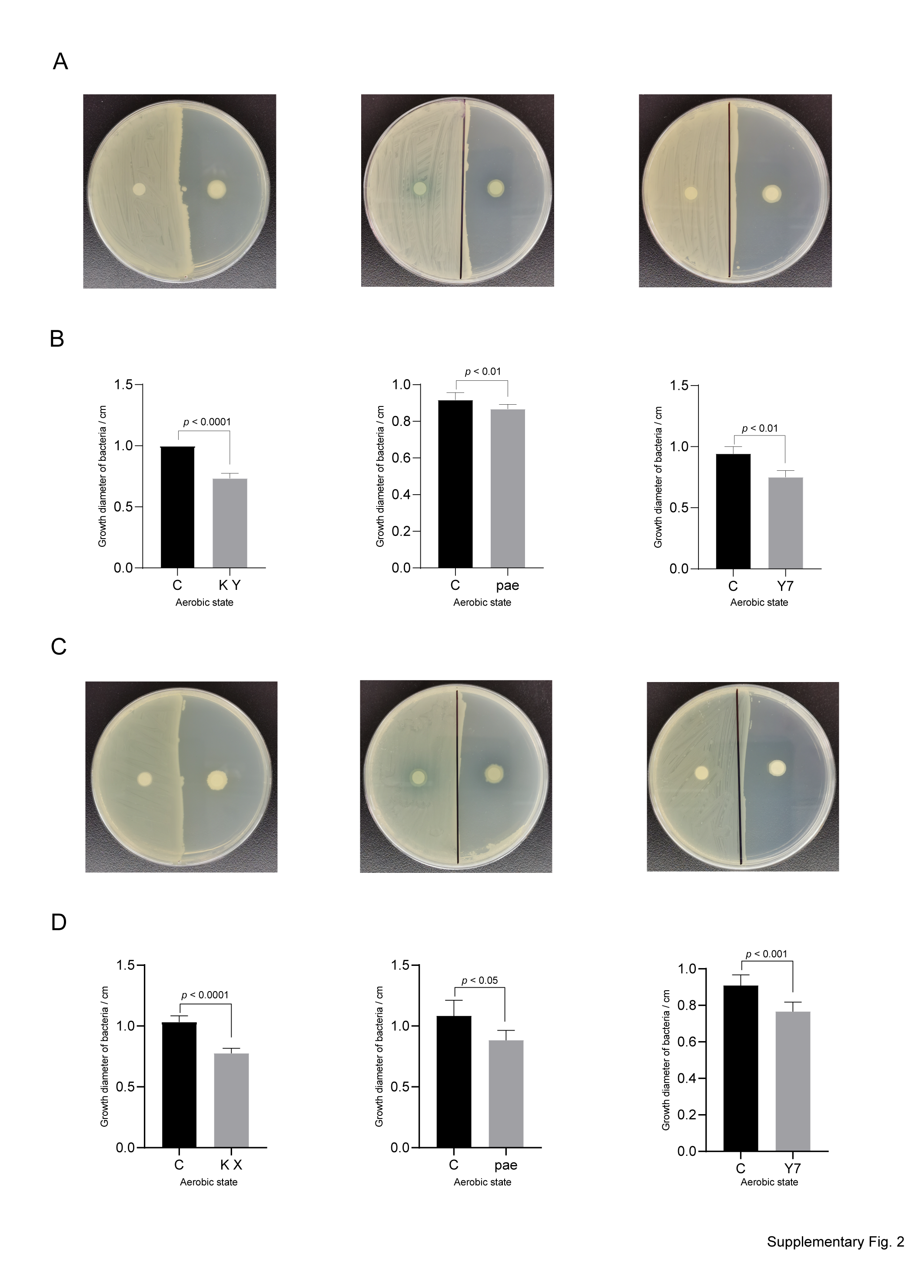
**

**Figure S2** | Antagonism experiment comparing *K. pneumoniae* KX/KY and cultivable bacteria in the housefly larval intestine in an aerobic environment. **(A)** Antagonism experiment comparing *K. pneumoniae* KX and cultivable bacteria, including *K. pneumoniae* KY, *Pseudomonas aeruginosa* and *Providencia stuartii*. KY: *K. pneumoniae* KY; pae: *P. aeruginosa*; Y7: *P. stuartii*. **(B)** Competitive inhibition between *K. pneumoniae* KX and cultivable bacteria in the housefly larval intestine. **(C)** Antagonism experiment between *K. pneumoniae* KY and cultivable bacteria, including *K. pneumoniae* KX, *P. aeruginosa* and *P. stuartii*. KX: *K. pneumoniae* KX; pae: *P. aeruginosa*; Y7: *P. stuartii*. **(D)** Competitive inhibition between *K. pneumoniae* KY and cultivable bacteria in the housefly larval intestine. Data are shown as the means ± SEMs. The t test was used for the statistical analysis.


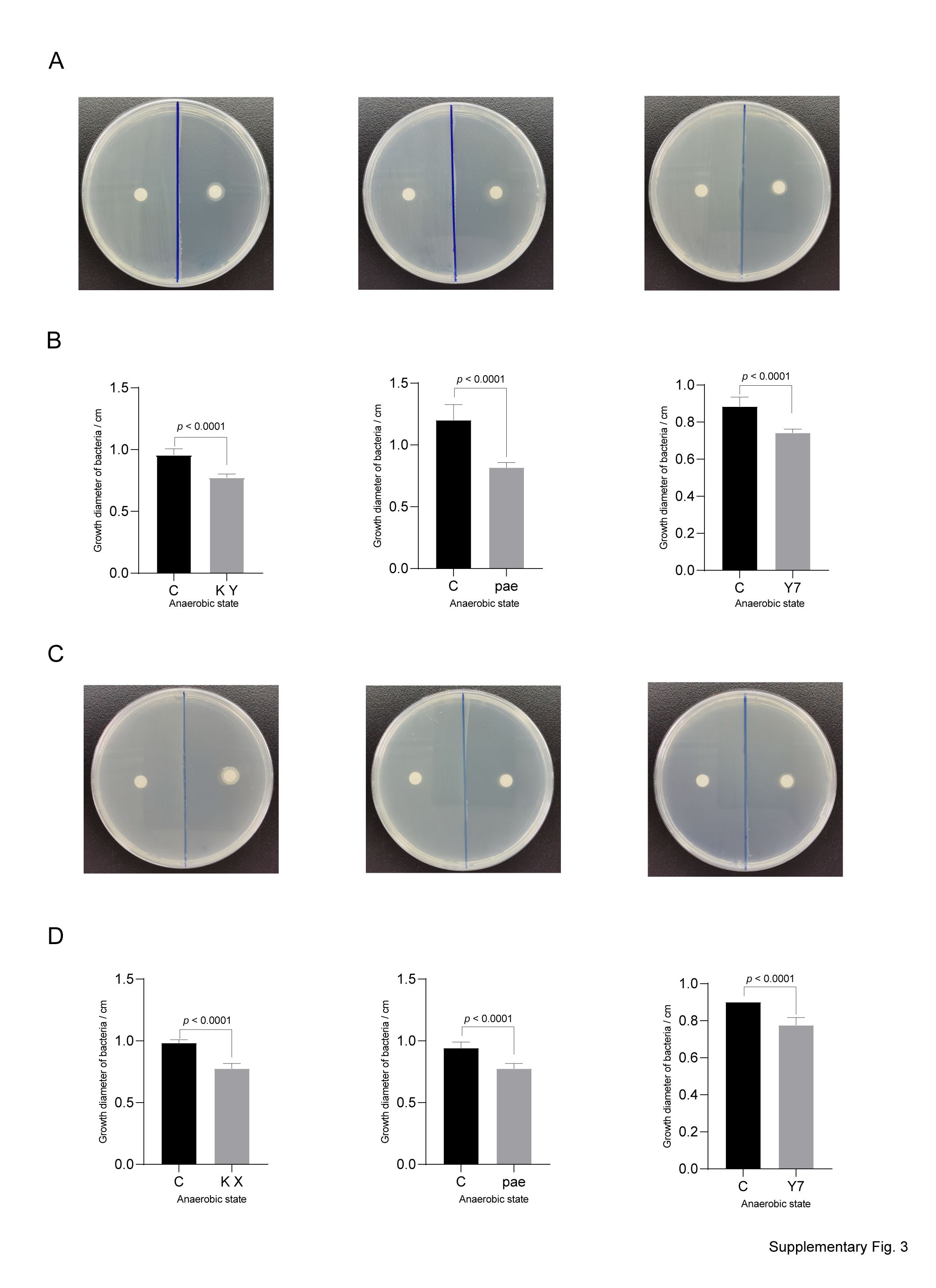


**Figure S3** | Antagonism experiment comparing *K. pneumoniae* KX/KY and cultivable bacteria in the housefly larval intestine in an anaerobic environment. **(A)** Antagonism experiment comparing *K. pneumoniae* KX and cultivable bacteria, including *K. pneumoniae* KY, *Pseudomonas aeruginosa* and *Providencia stuartii*. KY: *K. pneumoniae* KY; pae: *P. aeruginosa*; Y7: *P. stuartii*. **(B)** Competitive inhibition between *K. pneumoniae* KX and cultivable bacteria in the housefly larval intestine. **(C)** Antagonism experiment between *K. pneumoniae* KY and cultivable bacteria, including *K. pneumoniae* KX, *P. aeruginosa* and *P. stuartii*. KX: *K. pneumoniae* KX; pae: *P. aeruginosa*; Y7: *P. stuartii*. **(D)** Competitive inhibition between *K. pneumoniae* KY and cultivable bacteria in the housefly larval intestine. Data are shown as the means ± SEMs. The t test was used for the statistical analysis.
